# Laminar Wall Jet Flow and Heat Transfer over a Shallow Cavity

**DOI:** 10.1155/2015/926249

**Published:** 2015-08-30

**Authors:** P. Maheandera Prabu, K. P. Padmanaban

**Affiliations:** Department of Mechanical Engineering, SBM College of Engineering & Technology, Dindigul, Tamil Nadu 624 005, India

## Abstract

This paper presents the detailed simulation of two-dimensional incompressible laminar wall jet flow over a shallow cavity. The flow characteristics of wall jet with respect to aspect ratio (AR), step length (*X*_*u*_), and Reynolds number (Re) of the shallow cavity are expressed. For higher accuracy, third-order discretization is applied for momentum equation which is solved using QUICK scheme with SIMPLE algorithm for pressure-velocity coupling. Low Reynolds numbers 25, 50, 100, 200, 400, and 600 are assigned for simulation. Results are presented for streamline contour, velocity contour, and vorticity formation at wall and also velocity profiles are reported. The detailed study of vortex formation on shallow cavity region is presented for various AR, *X*_*u*_, and Re conditions which led to key findings as Re increases and vortex formation moves from leading edge to trailing edge of the wall. Distance between vortices increases when the step length (*X*_*u*_) increases. When Re increases, the maximum temperature contour distributions take place in shallow cavity region and highest convection heat transfer is obtained in heated walls. The finite volume code (FLUENT) is used for solving Navier-Stokes equations and GAMBIT for modeling and meshing.

## 1. Introduction

The cavity flow behavior is used in many engineering applications like solar energy systems, combustion chamber, and turbine blade tip flows. The flow simulation of wall jet over a shallow cavity is widely cited as the example envisioned of separated flow which would lead to deeper insight into the behavior of the nature of wall jet. Arous et al. solved the flow separation of wall jet flow and boundary layer flow over a shallow cavity for turbulent flow conditions [1]. Where FLUENT 6.3 was used for simulation and GAMBIT 2.3 was used as a preprocessor. The simulation had shown that of three eddy recirculation zones inside the cavity. Wall jet upstream reattachment lengths were relatively shorter than that of boundary layer flow. Sinha et al. found out the reattachment length of laminar separating flow over backward facing step for 100 < Re_*h*_ < 1000 experimentally [2]. Kanna and Das [3] numerically investigated the laminar wall jet flow under backward facing step for different step length and height using vorticity stream function. The recirculation, reattachment length, and flow characteristics of the wall jet were calculated. Barton discussed the backward facing step flow using SIMPLE algorithm numerically [4]. Five different schemes and various grid formations were compared based on reattachment *x*_1_, *x*_2_, and  *x*_3_. Barton solved entrance effect of various inlets upstream the flow over backward facing step (BFS) [5]. The shortest reattachment length and separation length were predicted and compared with experimental data for both uniform velocity inlet and parabolic velocity inlet profiles. Bajura and Szewczyk [6] carried out the experimental results for laminar wall jet flow profile at exit. The Reynolds numbers were taken from 270 to 770 and the similarity velocity profiles for different downstream location had been obtained. The experimental results were compared with theoretical results which indicated the stability of laminar plane wall jet flow. Seidel numerically found out the simulation of forced laminar and turbulent wall jet flow over the heated surface [7]. Selimefendigil and Öztop numerically found out the flow behavior of laminar pulsating flow in a backward facing step with adiabatic thin fin mounted top wall [8]. FLUENT software had been used to solve the governing equations. The heat transfer effect was increasing with increase in Reynolds number and with fin length fixed at top wall. The 188 percent of heat enhancement corresponding to 1.5*H* fin length obtained was compared with the same geometry at Re = 200 without fin setup. Kanna and Das simulated the flow pattern and characteristics of laminar offset jet with respect to offset ratio (OR) and Reynolds number (Re) [9]. *u*-velocity, streamline, vector plot, and wall vorticity were predicted in this paper. *u*-velocity was affected due to increase in Re and was not affected by OR. Gartling numerically studied the backward facing step flow using finite element Galerkin method [10]. The outflow boundary condition was used and influence evaluated at the separation of downstream flow. Armaly et al. observed the BFS flow distribution, velocity profile, and reattachment length by experimental and theoretical analysis from Reynolds numbers range 70 to 8000 [11]. The experimental results of laminar flow reattachment point were compared theoretically up to Re = 800. Chiang and Sheu [12] numerically evaluated the three-dimensional backward facing step flow with Armaly et al. [11] experimentally in the range of Re 100–1000. Zdanski et al. [13] numerically simulated the flow over shallow cavity by both laminar and turbulent cases. For the laminar cases, Re 147, 294, 442, and 662 were taken for simulation to find out the streamline, cavity floor pressure distribution, and also bubbles center to center (*x*_1_) distance for different aspect ratios using SIMPLER algorithm. Avelar et al. [14] studied the flow over a shallow cavity experimentally. The cavity streamlines were compared with Zdanski et al. [13] for aspect ratios 10, 8, and 6. Mesalhy et al. [15] studied *k*-*ε* turbulent flow and heat transfer effect on flow over a shallow cavity. The experimental and numerical results were compared based on the aspect ratio and Reynolds number. The local Nusselt number was mainly increased on the cavity floor due to the flow structure. Bhatti and Aung [16] analyzed the laminar forced convection heat transfer in rectangular cavity flow using the finite difference method. The effect of streamline behavior significantly developed the heat transfer effect in the upstream step. Shen and Floryan [17] simulated the rectangular cavity flow for the low Reynolds numbers. Stalio et al. [18] performed the numerical prediction of laminar flow and heat transfer of periodic rectangular cavities for low Prandtl number cases. The simulation was made in the Reynolds number range of 24.9–2260 and the Prandtl numbers 0.025 and 0.71 for investigation. The maximum global Nusselt number was achieved at AR 10 and Pr 0.71. Gresho et al. [19] proved that incompressible two-dimensional, steady state flow Navier-Stokes equations past a BFS at the Reynolds number 800 are stable at *t* → *∞*. In order to get higher accuracy, second-order upwind momentum equations were solved with coupled pressure-velocity. Huai et al. [20] used the second-order upwind discretization scheme for solving convective and diffusion terms. Pai et al. [21] simulated the effect of passing plug in chaotic mixing cavity. Sivasamy et al. [22] and Muthukannan et al. [23] have investigated laminar slot jet impingement flows with uniform velocity profile as inlet, whereas we investigate laminar wall jet flow with uniform inlet velocity profile. Arous et al. [24] analyzed interaction of jet and cavity in depth with turbulent flow.

The present objective of this research work represents the numerical simulation of laminar wall jet flow over a shallow cavity. The effect of various aspect ratios (AR), step length (*X*_*u*_), and Reynolds number (Re) in a shallow cavity under the wall jet flow condition will be investigated. The flow behaviors, streamline, vorticity formation, reattachment length, and upstream characteristics at leading edge and trailing edge are to be found based on AR and Re.

## 2. Problem Description and Mathematical Formulation

**Figure 1:**
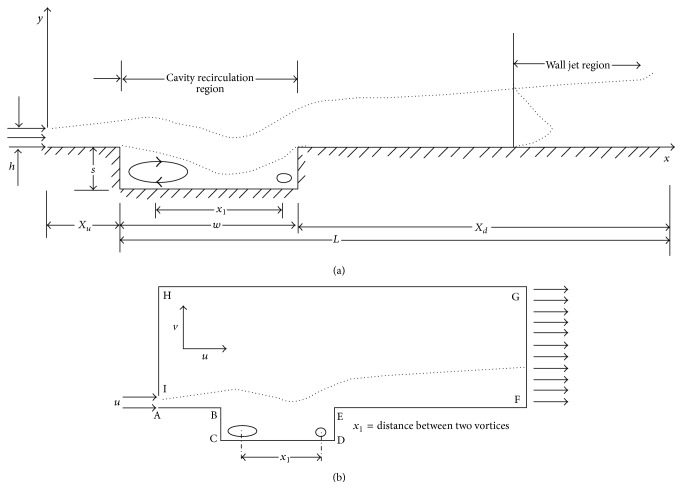
(a) Schematic diagram of physical problem. (b) Computational domain.

The schematic diagram of physical problem taken in this computational study is shown in Figures 1(a) and 1(b).

The two-dimensional incompressible laminar wall jet flow under the shallow cavity is taken into account. In general, the parabolic inlet velocity profiles are used in the numerical investigation of flow over shallow cavity. In this present work, the wall jet is assumed to be a uniform inlet velocity profile and the jet density is assumed to be atmosphere air density.

The governing equations for two-dimensional and incompressible laminar flow, mass, and momentum equations were expressed in the finite difference conservation form as follows.

*Continuity Equation*

(1) $$\begin{array}{c} \frac{\partial U}{\partial X}+\frac{\partial V}{\partial Y}=0. \end{array}$$

*Momentum Equations*

*x-Momentum*

(2) U∂U∂X+V∂U∂Y=−∂P∂X+1Re⁡∂2U∂X2+∂2U∂Y2.

*y-Momentum*

(3) U∂V∂X+V∂V∂Y=−∂P∂Y+1Re⁡∂2V∂X2+∂2V∂Y2.

*Energy Equation*

(4) $$\begin{array}{c} U\frac{\partial \theta }{\partial X}+V\frac{\partial \theta }{\partial Y}=\frac{1}{\text{Re Pr}}\left(\frac{\partial^{2}\theta }{\partial X^{2}}+\frac{\partial^{2}\theta }{\partial Y^{2}}\right). \end{array}$$

The uniform velocity inlet slot height (AI) is assumed to be 0.01, where, in Figure 1(b), AI is considered as jet inlet.

Based on no slip situation, *u* = 0 and *v* = 0 along AB, BC, CD, DE, EF, and HI.

Along GH,

(5) $$\begin{array}{c} \frac{\partial u}{\partial x}=0. \end{array}$$

Along GF, the fully developed flow condition,

(6) $$\begin{array}{c} \frac{\partial \phi }{\partial x}=0,\;\phi \left(u,v,p,\omega \right). \end{array}$$

Assume that the walls AB, BC, CD, DE, and EF are maintained at adiabatic (*θ*) 400 K.

Along the solid boundaries, *u* = 0 and *v* = 0.

The inlet fluid temperature is set as ambient at (*θ*) 303 K.

Computational channel height is (AH) = 20*h* and the flow length *L*, that is, AF = 40*h*, is found to be sufficient for all the cases. Aspect ratios (AR = w/s), AR = 1, 2, and 4, are used for simulation [16]. The step length (*X*_*u*_) is taken as *X*_*u*_ = 1*h*, 2*h*, 3*h* [3]. The stability of plane laminar wall jet flow is studied for the Reynolds number up to Re = 770 by Bajura and Szewczyk [6]. Gresho et al. [19] analyzed and proved the stability of laminar flow over backward facing step for Navier-Stokes equations range up to Re = 800. The low Reynolds numbers = 25, 50, 100, 200, 400, and 600 are taken for laminar wall jet flow over a shallow cavity simulation. In this present work, all the inlet velocities of Reynolds number are taken based on the hydraulic diameter of computational velocity inlet (*h*) domain. *X*_*d*_ is taken as downstream length of the computational domain. All the parameters are nondimensional.

## 3. Numerical Procedure

The commercial finite volume code FLUENT is used to solve continuity (1), *x*-momentum (2) and *y*-momentum equation (3), and energy equation (4) along with initial and boundary conditions (5), (6). In order to get higher accuracy, second-order upwind momentum equations are solved with coupled pressure-velocity. Qiu et al. [25] used the second-order upwind discretization scheme with SIMPLEC algorithm for solving convective and diffusion terms. At present, the third-order QUICK scheme is used for solving momentum equations and SIMPLE algorithm for couple pressure-velocity. The convergence limits for mass and *x*- and *y*-momentum residuals are 10^−4^ and 10^−5^, respectively.

## 4. Validation

**Figure 2:**
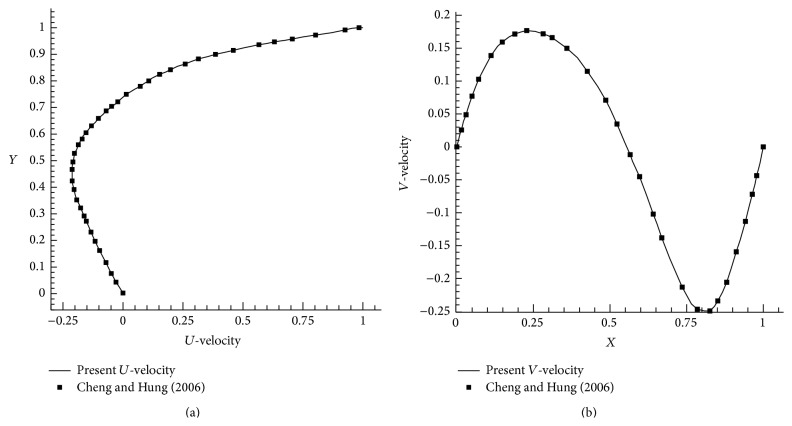
(a) *U*-velocity, Re = 100, grid 129 × 129. (b) *V*-velocity, Re = 100, grid 129 × 129.

**Figure 3:**
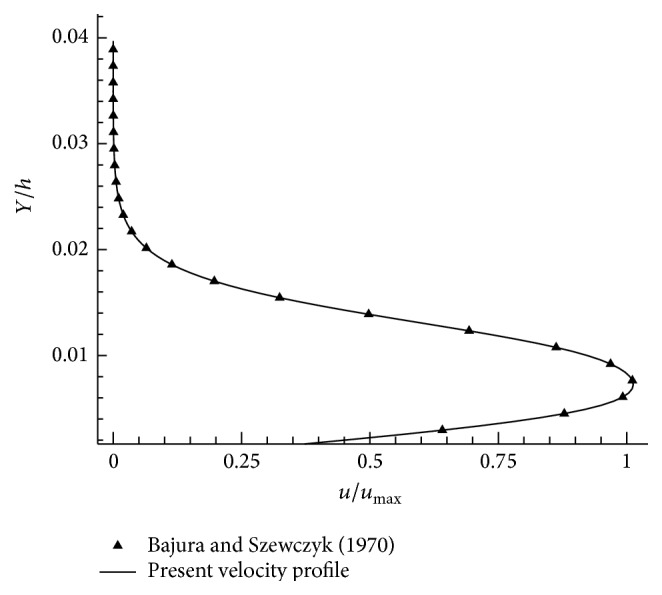
Plane wall jet velocity profile (*u*/*u*_max⁡_), Re = 752, *X*/*L* = 180.

To validate the numerical procedure, the lid driven cavity flow, wall jet flow over a flat plate, and backward facing step problems have been solved. Cheng and Hung [26] solved lid driven rectangular cavity flow for Reynolds number = 100. We have compared *U* and *V* velocities in Figures 2(a) and 2(b) and the similar velocity profiles have been obtained. Initially, Glauert [27] explained and predicted the laminar velocity profile in flow behavior of wall jet. Bajura and Szewczyk [6] experimentally investigated the laminar plane wall jet flow for the Reynolds numbers in the range of Re = 270–770 and the peak velocity position has been calculated. This experimental result has been taken into account in the case of wall jet flow over a flat plate. The peak velocity of the wall jet is compared with experimental results for Re = 752 and *X*/*L* = 180 and it is found to be in good agreement with experimental results as shown in Figure 3.

**Figure 4:**
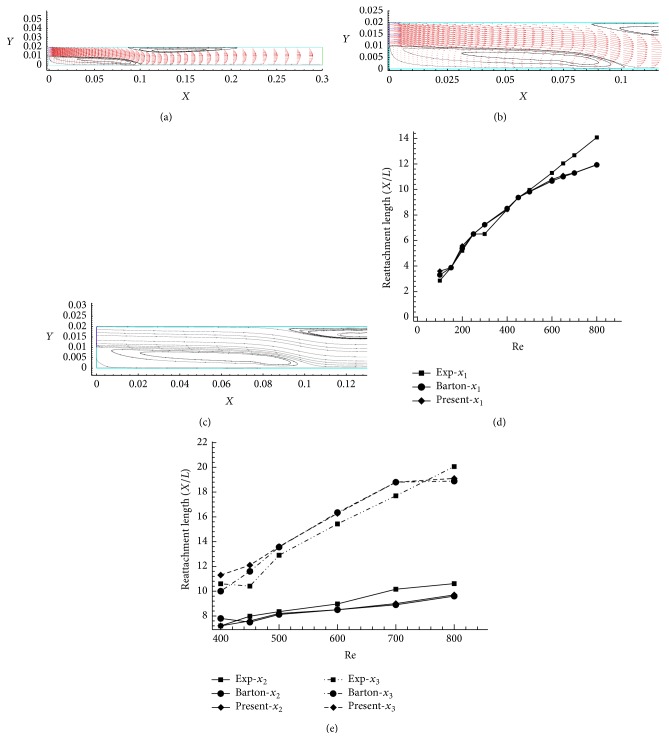
(a) BFS uniform inlet velocity, vorticity formation, streamline, and vector plot for Re = 500. (b) Reattachment length (*x*_1_) and separation length (*x*_2_) for uniform velocity inlet profile, streamline, and vector plot for Re = 500. (c) BFS streamline and reattachment point for Re = 800. (d) *x*_1_: reattachment length (*X*/*L*) for various Re. (e) Separation length (*x*_2_), separation reattachment length (*x*_3_) for various Re.

Fluid flow over the backward facing step is an excellent benchmark problem to simulate the sudden expansion flow and the reattachment flow. Barton [4] numerically calculated the reattachment length and separation lengths for different low Reynolds numbers (Re = 50–600). The inlet velocity profile plays an important role in the sudden spreading-out flow problems. Barton [5] had compared the results of the backward facing step flows with and without entrance regions; the semi-implicit method for pressure linked equations (SIMPLE) algorithm was used for flow simulation. The same simulation has been done and the reattachment point (*x*_1_) as shown in Figure 4(b) and separation points (*x*_2_  and  *x*_3_) as shown in Figures 4(b) and 4(c) are compared.

Figure 4(a) shows the eddy formation and vector plot of backward facing step flow with uniform velocity inlet profile for Re = 500. Figures 4(b) and 4(c) indicate the reattachment length (*x*_1_) and separation length (*x*_2_) simulated for Re = 500 and Re = 800. The reattachment length (*x*_1_), separation length (*x*_2_), and separation reattachment length (*x*_3_) have been compared for various Re (Figures 4(d) and 4(e)). These numerically calculated values are compared with numerical and experimental results available in open literature and are found to be in good agreement with them.

## 5. Grid Independent Study

The uniform clustered grid points are used nearer to the slot height (*h*). The grid test for AR = 1, *X*_*u*_ = 1, and Re = 200 is carried out.

**Figure 5:**
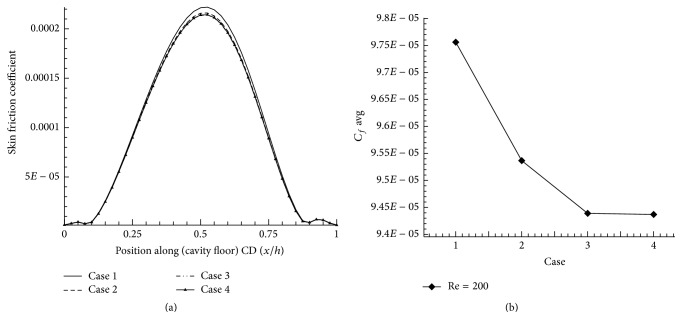
(a) Skin friction coefficient on cavity floor. (b) Average skin friction coefficient on cavity floor.

**Figure 6:**
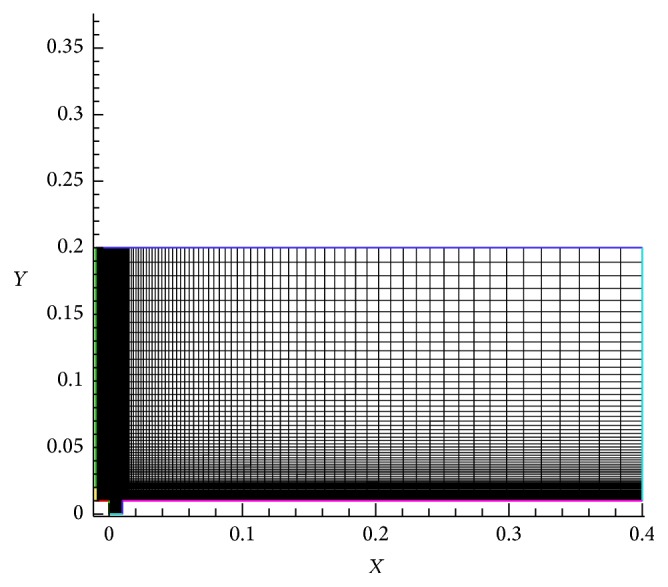
Typical grids for computation.

Number of grid independent study have been done and based on the skin friction coefficient (*C*_*f*_), optimized grids are considered for computation; average skin friction coefficient (*C*_*f*_ avg) of cavity floor region for Re = 200 (Figures 5(a) and 5(b)) is obtained. The quadrilateral cells ranges from 13300 to 18600 for grid independent study. The grid study is shown in Table 1 by approximately 40% increases in number of cells. The skin friction coefficient deviation is found out to be 0.02% which corresponds to case  3. This is the least error % which is less than 1%. It can be indicated that case  3 is having the optimum grid points. The typical computational geometry is shown in Figure 6.

## 6. Results and Discussion

In this present work, we deal with the results of laminar wall jet flow over a shallow cavity. The key parameters that we have changed are aspect ratio of cavity (AR = 1, 2, and 4), step length (*X*_*u*_ = 1 to 3), and Reynolds number (25, 50, 100, 200, 400, and 600). The air viscosity 1.6*E* − 05 kg/m-s has been chosen based on the air temperature of the wall jet. The uniform inlet velocity profile passes from the air inlet slot (*h*), extends to the flat wall, and expands to shallow cavity. Our objective is to predict the effect of AR, *X*_*u*_, and Re in the wall jet flow over a shallow cavity.

### 6.1. Effect of Reynolds Number (Re)

**Figure 7:**
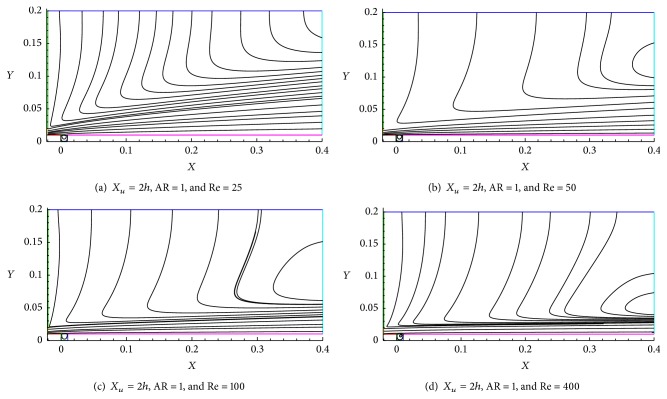
Streamline formation for Re = 25, 50, 100, and 400.

**Figure 8:**
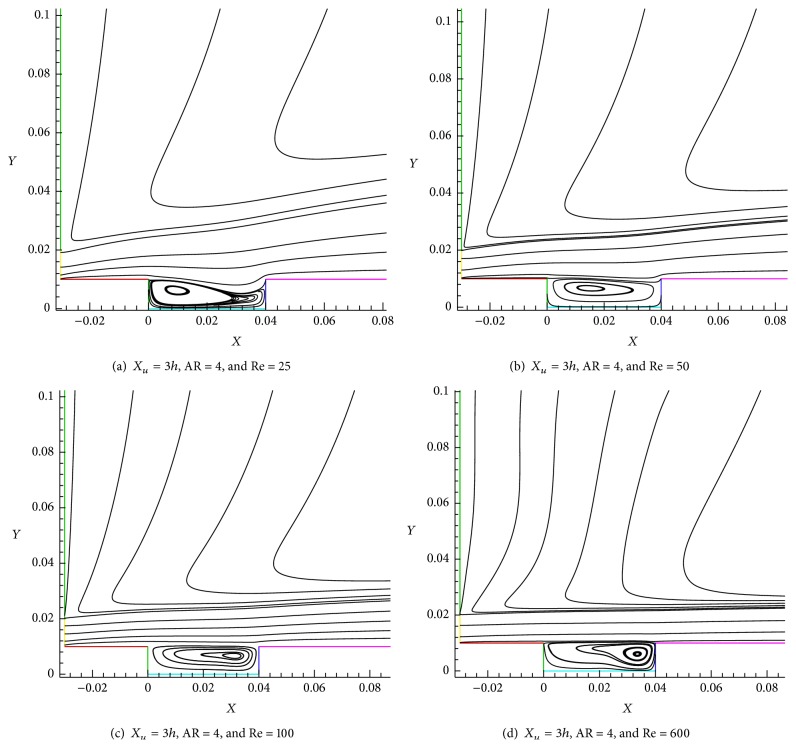
Recirculation of the shallow cavity for different Re = 25, 50, 100, and 600.

**Figure 9:**
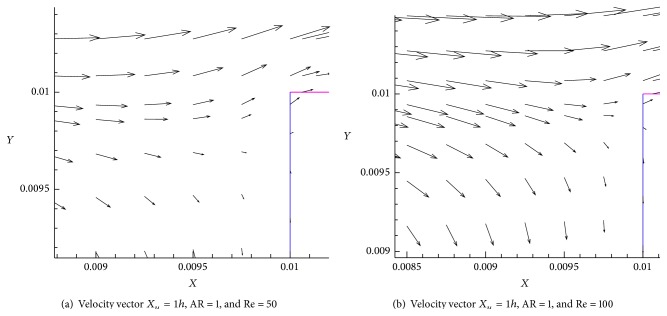
Velocity vector for *X*_*u*_ = 1*h*, AR = 1, and Re = 50 and 100.

**Figure 10:**
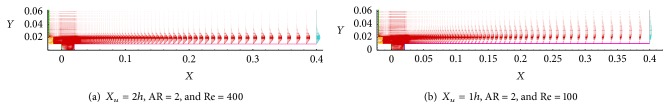
Velocity vector plot of the wall jet shallow cavity.

The effect of Reynolds number in shallow cavity flow topologies is shown in Figures 7–10. The structure of the wall jet flow over a shallow cavity might be easily discriminated by the reflection on the streamline of fluid flow as illustrated in Figures 7(a)–7(d).  Figure 7(a) shows that the streamlines seem to be parallel at exit, which indicates that the fully developed flow occurs at the end of the computational domain.

A similar kind of streamline profile was obtained by Kanna and Das [3]. When Reynolds number increases from Re = 25 to 400, the flow is detained near to the wall region (Figures 7(a) and 7(b)) and the wall jet spreads over a larger area for low Re and lesser area for higher Re (Figures 7(c) and 7(d)); this indeed is due to shear force of the fluids.

The recirculation flow is formed in the shallow cavity region (Figure 1(a)) as shown in Figures 8(a)–8(d). The development of strong eddy downstream backward facing step is due to the results of geometrical effect as well as the flow nature of wall jet. We can observe the vortex formed almost near the left corner of the shallow cavity as shown in Figure 8(a). We can view the size of the vortex by the increasing from Re = 50 to Re = 100 (Figures 8(a) and 8(b)). The center of the recirculation is moved from left side of wall (BC) into right side of wall (DE) of the shallow cavity (Figures 8(b) and 8(c)). When Reynolds number is increased from Re = 100 to Re = 600, in Re = 600 (Figure 8(d)), the shape of the central recirculation vortex is found to be petite than Re = 100 (Figure 8(c)) and seen to move towards the side wall (DE). It can be seen that the Reynolds number performs significant factor in the formation of vortex in wall jet flows over a shallow cavity.

Figure 9 shows the velocity vectors for various Re flows in the cavity region. We can see that all the Re cases generate a single governing eddy within the downstream segment of the shallow cavity and also a no disturbance to the mean flow on the upstream shallow cavity (Figures 9(a) and 9(b)). The shear layer is formed inside the cavity for low Re (Re = 50) and shear layer is formed at the top of the cavity for higher Re (Re = 100). It occurs due to the domination of the eddy at shallow cavity. When Re increases domination of eddy inside shallow cavity also increases. As we see in Figure 10(a) the leading edge velocity vector and trailing edge velocity vector have parallel component to fluid flow direction. The fully developed wall jet flow vector velocity profile is observed at the end of the computational domain (Figures 10(a) and 10(b)). It is specified that this computational domain (length and height) is sufficient for analysis. We found that the larger vector velocity profile is obtained at Re = 100 (Figure 10(b)) compared to Re = 400 (Figure 10(a)). It clearly indicates that when Re increases, the size of the vector velocity profile decreases.

**Figure 11:**
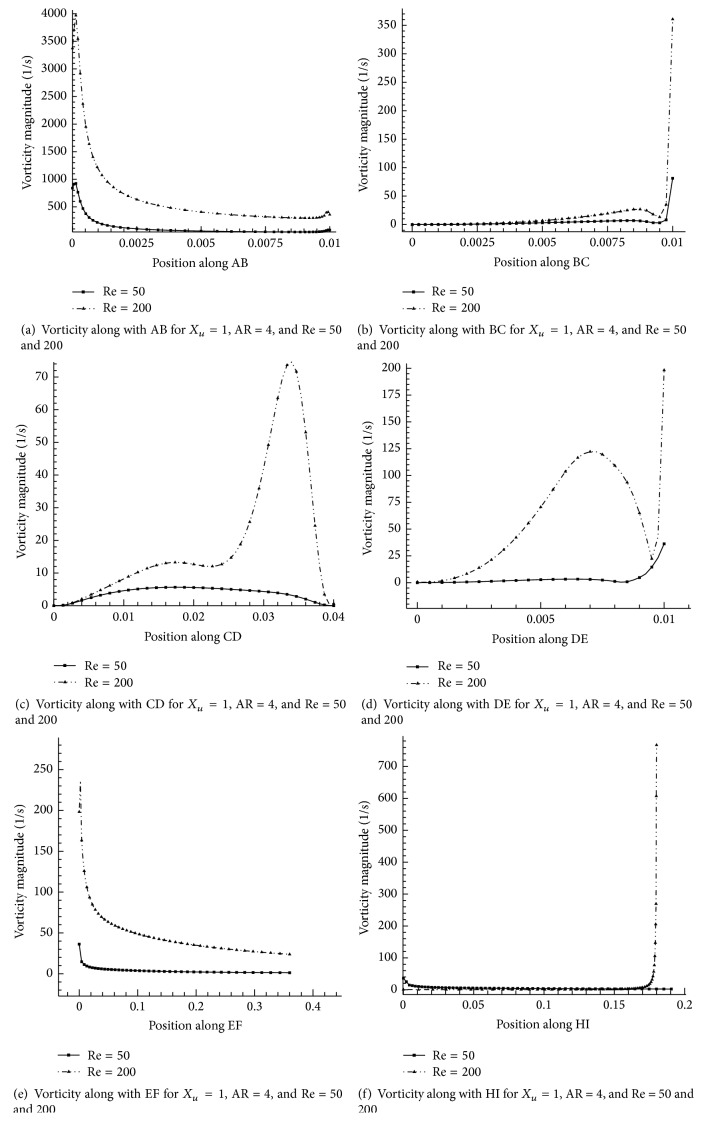
Effect of Reynolds number on wall surface vorticity: *X*_*u*_ = 1*h*, AR = 4.

Walls are source for vorticity. The vorticity is the major phenomenon to be considered in shallow cavity flow domain. It is assumed that in this present computational flow domain AB, BC, CD, DE, EF, and HI are walls.  Figure 11 shows the Re effect in walls surface (AB, BC, CD, DE, EF, and HI). The influence of Re on vorticity along position AB is shown in Figure 11(a). It can be absorbed from the figure that *u*-velocity gradient in the normal direction is increased. The local maximum vorticity magnitude is reached at *x*/*h* = 0.013 for both Re = 200 and 50 cases shown in Figure 10(a). When Re increases the magnitude efficiency is lesser and similar results were obtained by Kanna and Das [3].  Figure 11(b) shows the vorticity behavior on position BC. It can be seen that when Re increases the vorticity magnitude increases. In Figure 11(c), it is observed that vorticity magnitude reaches the lowest value when Re = 50 and reaches the maximum value at *x*/*h* = 3.52 for Re = 200. It is clearly observed from this figure that when Re increases, the vorticity magnitude gradually increases.  Figure 11(d) indicates the effect of Re at wall surface DE. It is seen that there is no fluctuation of vorticity magnitude for Re = 50 and it is slowly increased up to the corner position (*x*/*h* = 1). We can see the variation of vorticity magnitude for Re = 200 particularly near the corner point. When Re increases, the effectiveness of the vorticity magnitude will be less significant [28] at corner of the wall.  Figure 11(e) shows the effect of Re at surface EF. It is noticeable that the vorticity magnitude decreases slowly up to the end of the wall for both Re = 50 and Re = 200. The two parallel vorticity magnitude lines (*x*/*h* = 40) indicate the effect of Re and fully developed flow at end of computational domain. Vorticity variation on wall HI is shown in Figure 11(f). The maximum vorticity magnitude is reached at *x*/*h* = 18 for both cases (Re = 50 and 200). Low Re creates less vorticity at the corner and vice versa.

### 6.2. Effect of Aspect Ratio (AR)

**Figure 12:**
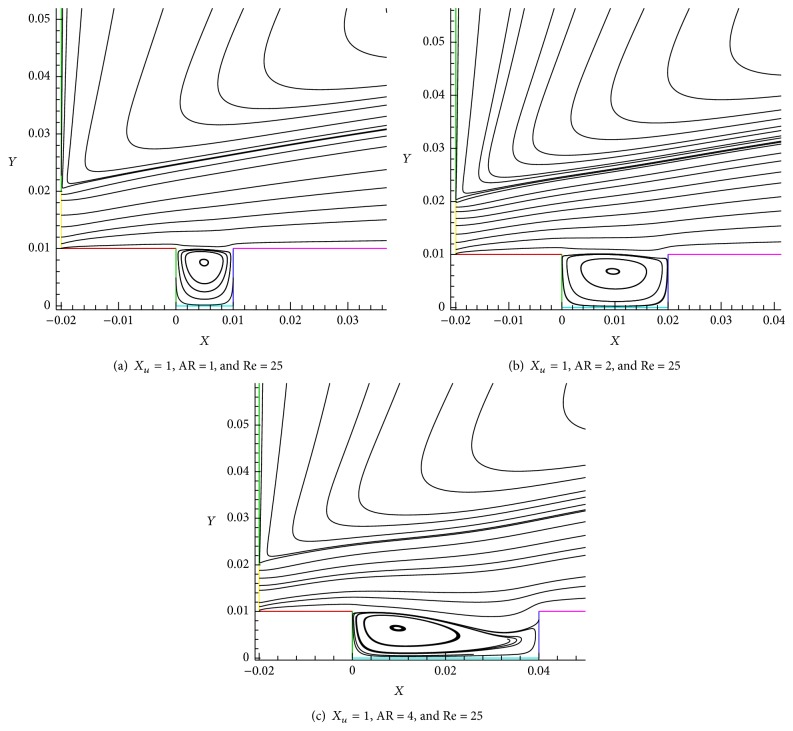
Effect of aspect ratio at shallow cavity region.

**Figure 13:**
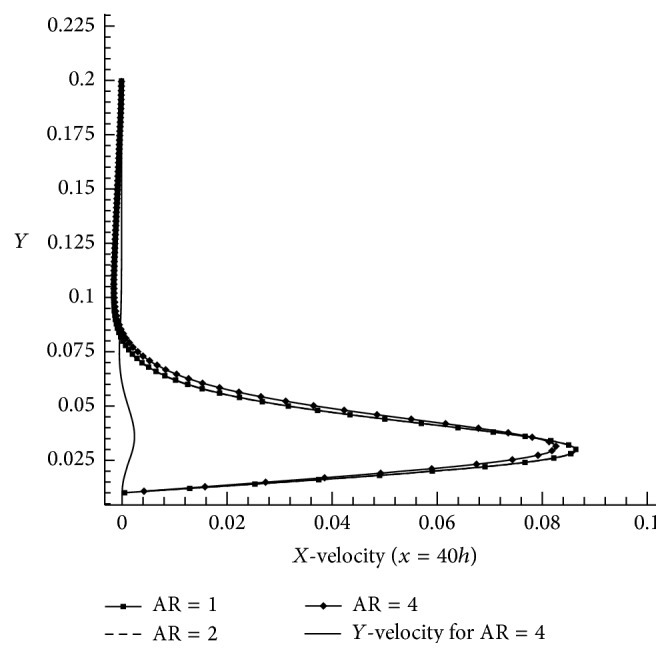
Effect of aspect ratio at exit velocity for *X*_*u*_ = 1, Re = 100, and AR = 1, 2, and 4.

**Figure 14:**
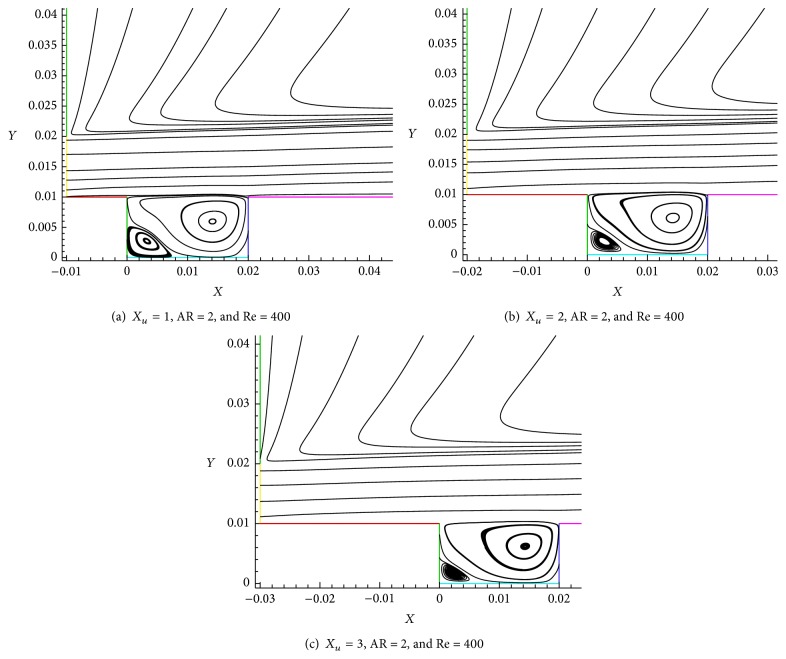
Effect of step length on shallow cavity vortex formation for AR = 2, Re = 400, and *X*_*u*_ = 1, 2, and 3.

Figures 12, 13, and 14 show the influence of aspect ratio in shallow cavity region. The Reynolds number (Re) 25 is taken as constant (Figures 12 and 13). It is observed that different vortex formations are formed which are shown in Figures 12(a), 12(b), and 12(c) we can observe the single vertex formed inside of the shallow cavity region (Figure 12(a)). Similarly, formation of single vortex has been observed (AR = 1, AR = 2) by Shen and Floryan [17] for low Re with the boundary layer flow condition. In the wall jet flow case discussed in this paper, we obtained one strong vortex which is (streamline) spread in all the sides of cavity.

Figure 12(b) shows that there is a vortex formation at center of the shallow cavity for AR = 2. In this wall jet flow streamline splits along curved shape and is immersed into the cavity to the full height of the cavity. But in the case of AR = 3 (Figure 12(c)) the streamline is divided at 95% of the step height (*h*) on trailing edge and 80% of *h* on leading edge above the bottom of the cavity at the lowest streamline point. The one vortex formation is seen to be *x*/*h* = 0.95 and is almost 1/3 of cavity floor length (*w*). The effect of aspect ratio at the exit velocity is illustrated in Figure 13. *X*_*u*_ = 1 and Re = 25 are taken as constant values and AR (1, 2, and 4) effect is predicted. When we compare the exit velocities (*x*/*h* = 40) of AR = 1 and AR = 2 the difference amount is less with respect to two aspect ratios but at AR = 4 there is significant velocity change as compared to the former. It obviously indicates that the aspect ratio considerably acts as an important factor in shallow cavity flow under wall jet flow condition.

### 6.3. Effect of Step Length (*X*_*u*_)

The influence of step length (cases *X*_*u*_ = 1, *X*_*u*_ = 2, and *X*_*u*_ = 3) is clearly observed (AR = 2 with Re = 400). Figures 14(a), 14(b), and 14(c) indicate the streamline velocity and vortex formation for different step lengths in the shallow cavity region. It can be seen easily in Figure 14(a) that there are two vortices formed at this juncture. The strong (large) vortex is formed at the leading edge (right side) and spreads into the cavity and the small vortex is generated at trailing edge (left side) at bottom of the cavity. The size of the vortex is gradually reduced for increase in step length (Figure 14(a) (*X*_*u*_ = 1, AR = 2, and Re = 400) and Figure 14(b) (*X*_*u*_ = 2, AR = 2, and Re = 400)). This is due to the increased step length.  Figure 14(c) shows that leading edge vortex dominates the vortex formed at bottom of trailing edge which is small in size.

**Figure 15:**
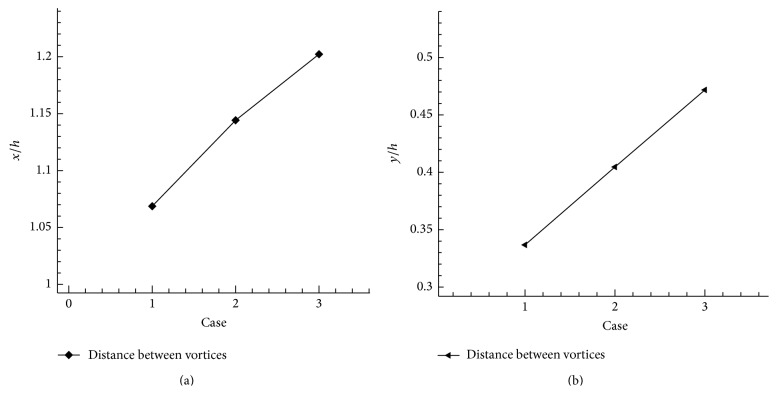
(a) Distance between two vortices on shallow cavity along *x*-coordinate. (b) Distance between two vortices on shallow cavity along *y*-coordinate.

**Figure 16:**
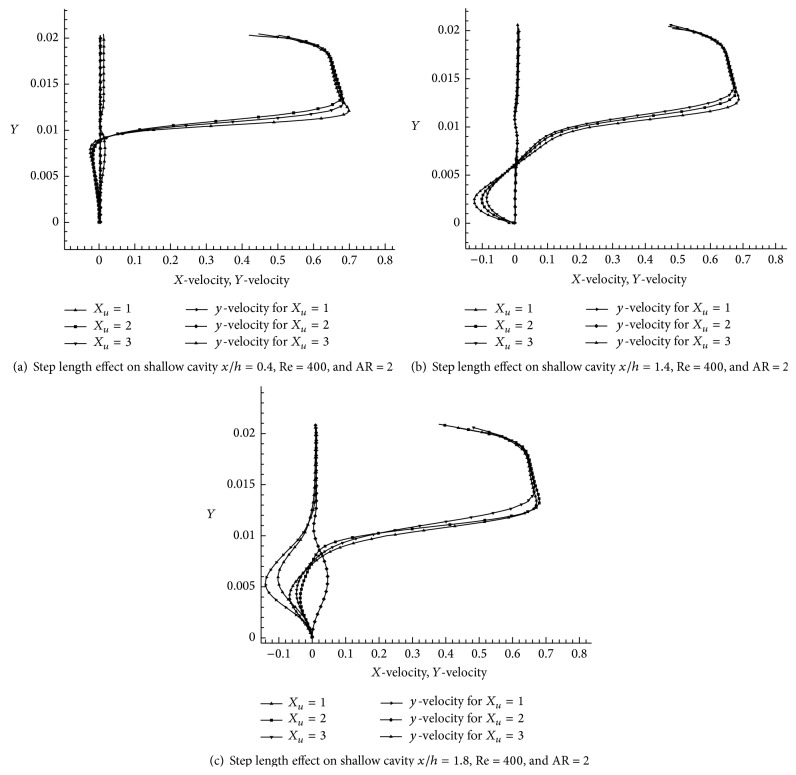
Velocity fluctuation due to influence of step length on shallow cavity.

The distance between the two vortices on shallow cavity region along *x*-coordinate and *y*-coordinate is shown in Figures 15(a) and 15(b). The distance for case *X*_*u*_ = 1 and case *X*_*u*_ = 2 is *x*/*h* = 1.068682 and *x*/*h* = 1.144188 and for *X*_*u*_ = 3 it is *x*/*h* = 1.202249, respectively, along *x*-coordinate. Similarly, along *y*-coordinate the maximum distance between the two vortices is achieved for case *X*_*u*_ = 3 (Figure 15(b)). From the results we conclude the distance between vortices when increased in step length. Hence step length is one of the factors that plays an important role in wall jet flow condition, the velocity fluctuation on cavity region is found out by varying step length which are shown in Figures 16(a), 16(b), and 16(c). The *x*-velocity variation for the cases *X*_*u*_ = 1, *X*_*u*_ = 2, and  *X*_*u*_ = 3 at *x*/*h* = 0.4 is plotted in Figure 16(a). The maximum *x*-velocity is attained from case *X*_*u*_ = 1 and the least *x*-velocity at case *X*_*u*_ = 3. We can see that the *x*-velocity is gradually decreased while increasing the step length as shown in Figures 16(b) and 16(c). The negative *x*-velocity is larger when step lengths are increased. It is seen (Figure 16(c) (*x*/*h* = 1.8)) that *y*-velocity is not stable and *x*/*h* = 0.4 and  *x*/*h* = 1.4 are more or less stable along *y*-direction.

## 7. Heat Transfer

**Figure 17:**
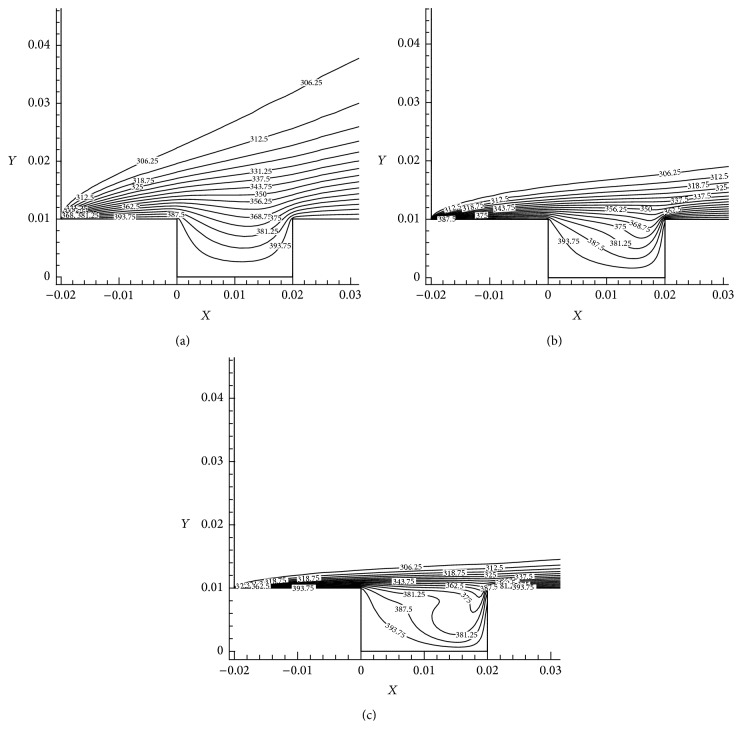
Temperature contours AR = 2, *X*_*u*_ = 2, and Re = 25, 100, and 400.

The various heat transfer outcomes had been shown based on the local Nusselt numbers (Nu). The wall jet flow effect with different Re is predictable to have enormous conflict on heat transmit results. The positions, where flow on a heated plate (AB, EF) and vortices are structured inside cavity (BC, CD, and DE), have greatest impact on heat transfer analysis for different Re cases. The walls (AB, BC, CD, DE, and EF) are maintaining uniform wall temperature. The temperature distribution contour and heat transfer in terms of Nu have been observed. Figures 17(a)–17(c) shows that the temperature contours of the shallow cavity for Re = 25, 100, and 400.

**Figure 18:**
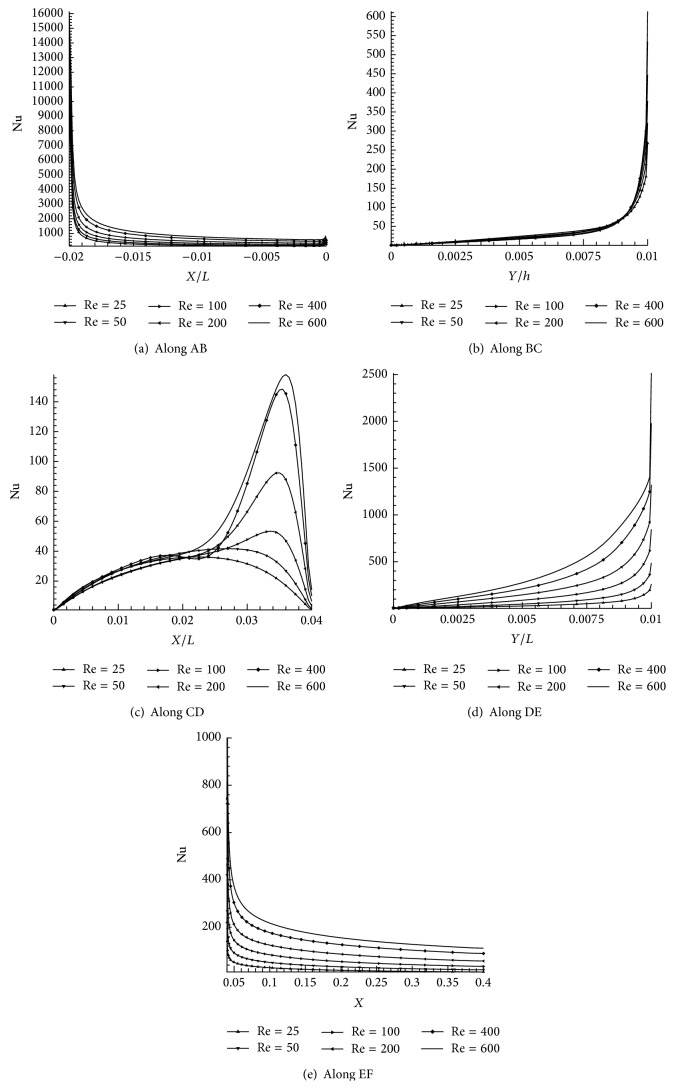
Distribution of Nu along AB, BC, CD, DE, and EF for Re = 25, 50, 100, 200, 400, and 600.

Figure 17(a) shows that the temperature contours are spread inside the cavity regime. It is comparatively lesser area than Figures 17(b) and 17(c). It is seen that the temperature contour lines move towards the bottom wall which occurs due to fluid shear forces. It clearly indicates that temperature distribution inside shallow cavity is increased when Re increases. Figures 18(a)–18(e) show the effect of Re in shallow cavity heat transfer observed by means of Nu.  Figure 18(a) shows the Nu variation along AB wall and the highest Nu has been observed for Re = 600. It can be seen that the Nu along wall BC is not having big variation due to Re as shown in Figure 18(b). The cavity floor (CD) heat transfer effect due to Re is shown in Figure 18(c). It has enormous fluctuation in Nu particularly at the end of the wall CD for the cases Re = 100, 200, 400, and 600 which happened due to reattachment effects. The maximum Nu is attained at *X*/*L* = 0.038 for the case Re = 600. It can be concluded that heat transfer is increased when Re increases.  Figure 18(d) indicates the heat distribution in terms of Nu along the wall DE. Nu is more at the leading edge due to higher velocity gradient (Nu is gradually increases when Re is increased). Similarly Figure 18(e) shows the Nu variation in wall EF due to increase in Re and the wall leading edge is having higher Nu. Finally it is concluded that the maximum heat transfer (Nu) is possible when Re increases.

## 8. Conclusion

In this present work, two-dimensional laminar wall jet flow over a shallow cavity is investigated numerically. The influences of various physical parameters (AR, *X*_*u*_) and flow parameter (Re) on the wall jet fluid flow over a cavity are determined by the solutions of governing equation. The fluid flow behavior characteristics like streamline, vortex (recirculation) formation, wall vorticity, velocity profile, and vector contour are examined for various Re, AR, and  *X*_*u*_. The results show that when Re increases (Re 25 to 100), the vortex moves from trailing edge wall side to leading edge wall side. In general, the wall vorticity gradually increases on all wall surfaces but rapidly increases on cavity floor and on leading edge wall surface with increase in Re. The center of the vortex is found to move from the center of the cavity floor to trailing edge wall side (left side) and the exit velocity of computational domain (FG) slowly decreases when AR is increased. When *X*_*u*_ decreases the distance between two vortices is reduced. The velocity profile along the length of the cavity bottom has been computed and was found not to considerably decrease as step length is increased. When Re increases, the maximum temperature contour distributions take place in shallow cavity region and highest convection heat transfer is obtained in heated walls.

## Figures and Tables

**Table 1:** Grid independence study.

Cases	Number of quadrilateral cells	Average skin friction coefficient for the cavity floor *C*_*f*_ × 10^−3^
Case 1	13300	0.09760
Case 2	14400	0.09537
Case 3	15200	0.09439
Case 4	18600	0.09437
